# Increased inter-atrial and intra-atrial conduction times in pediatric patients with non-alcoholic fatty liver disease

**DOI:** 10.1007/s00431-024-05809-8

**Published:** 2024-10-22

**Authors:** Doaa El Amrousy, Heba EL Ashry, Sara Maher, Yousef Elsayed, Karim Elkashlan, Dina Abdelhai, Wegdan Mawlana, Samir Hasan

**Affiliations:** 1 Pediatric Department, Faculty of Medicine, Tanta University, Tanta, Egypt; 2 Tropical Medicine Departments, Faculty of Medicine, Tanta University, Tanta, Egypt; 3 Theodor Bilharz Research Institute, Cairo, Egypt; 4 Faculty of Medicine, Cairo University, Cairo, Egypt; 5 Faculty of Medicine, Alexandria National University, Alexandria, Egypt; 6 Clinical Pathology Department, Faculty of Medicine, Tanta University, Tanta, Egypt

**Keywords:** NAFLD, AF, Children, Adolescents

## Abstract

The global incidence of pediatric non-alcoholic fatty liver disease (NAFLD) is rising, and it is linked to various potentially dangerous complications such as cardiovascular complications, particularly atrial fibrillation (AF). Atrial electromechanical conduction delay (EMD) has been reported as an early predictor for AF development. This study aimed to explore the link between NAFLD and the increased risk of AF development. This cross-sectional study was performed on 100 newly diagnosed NAFLD children (aged 14–18 years) as the patient group and 100 healthy individuals as a control group. Transthoracic echocardiography and simultaneous electrocardiography (ECG) recording were employed to estimate atrial electromechanical properties. EMD values were calculated for the inter-atrial, left intra-atrial, and right intra-atrial. Our results showed that pediatric patients with NAFLD exhibited significantly prolonged EMD values in the left and right intra-atrial as well as in inter-atrial regions compared to the control group (*P* = 0.03, *P* < 0.001, *P* < 0.01, respectively).

*Conclusion*: Children with NAFLD show atrial electromechanical alterations that may presage AF in adulthood.

**What is known:**

*• The global incidence of pediatric non-alcoholic fatty liver disease (NAFLD) is rising, and it is linked to various potentially dangerous complications such as cardiovascular complications, particularly atrial fibrillation (AF).*

*• Atrial electromechanical conduction delay (EMD) has been reported as an early predictor for AF development.*

**What is new:**

*• Children with NAFLD show atrial electromechanical alterations that may presage the appearance of AF in adulthood.
*

*• These children require multidisciplinary medical care to control liver disease and cardiovascular complications.*

## Introduction

In children, pediatric non-alcoholic fatty liver disease (NAFLD) stands as the extremely frequent persistent liver disease. The incidence of this disease has increased alongside the rising rates of obesity [1]. NAFLD extends beyond liver involvement and manifests as a multisystem disease, affecting various other organs. Its long-term effects persist throughout the adult years, resulting in substantial mortality and morbidity [2, 3].

Numerous studies have indicated a strong linkage between cardiovascular diseases (CVD) and NAFLD, signifying the NAFLD importance as a noteworthy standalone contributor for clinical and subclinical CVD, even if the standard metabolic syndrome and cardiovascular risk factors are not evident [4, 5]. NAFLD showed an increased risk of cardiovascular complications like cardiac arrhythmia, coronary artery disease (CAD), subclinical atherosclerosis, and conduction abnormalities [6]. The additional burden of NAFLD-related CVD intensifies the elevated cardiovascular morbidity and death in adulthood [7]. The etiology of fatty liver disease involves multiple factors, including genetics, intestinal dysbiosis, adipokines, inflammation, oxidative stress, and psychological stress like depression and anxiety [8, 9], all of which are recognized as indicators of CVD.

AF is the most frequently encountered arrhythmias associated with significant mortality and morbidity. The inflammation linked to NAFLD has been implicated in cardiac remodeling and the development of AF [10–13]. In a prospective study conducted in Finland, researchers observed a significant independent linkage between NAFLD and the incidence of AF. The increased risk of developing AF remained even after considering possible confounders, like diabetes status, body mass index (BMI), age, and gender [14]. Consistency of these findings across various large population-based cohorts has been variable. The Study of Health in Pomerania and the Framingham Heart Study indicated no substantial correlation among AF and either ultrasound-diagnosed hepatic steatosis or computerized tomography (CT) [15, 16].

Atrial electromechanical delay (EMD) has been reported in several studies as a good predictor for atrial arrhythmias especially AF in several diseases [17–19]. Limited data is available regarding the relationship between the risk of arrhythmias and NAFLD in pediatric populations. Hence, our study aimed to assess the atrial electromechanical conduction delay in children with NAFLD.

## Subjects and methods

This cross-sectional study was conducted at the Pediatric and Gastroenterology Departments, Tanta University Hospital, from March 2023 to September 2023. The study was approved by the local ethical committee of the Faculty of Medicine, Tanta University, with ethics approval code 36264PR277/23. Written informed consents were signed by the parents of all included children.

The study included a total of 100 consecutive newly diagnosed obese patients (14–18 years old) with NAFLD as the patient group. The control group comprised 100 healthy matched volunteers for age, sex, and body weight who visited our outpatient clinics for upper respiratory tract infections. These individuals were included in the study after the complete resolution of their infections.

Exclusion criteria: subjects with documented congenital or acquired heart disease, arrhythmias, use of anti-arrhythmic drugs for any reason, diabetes mellitus (DM), active infection, hypertension, organ failure, Cushing syndrome, hyperthyroidism, or hypothyroidism.

### Methods

#### Electrocardiography (ECG) analysis

After resting for 10 min, 12-lead ECG readings were recorded on paper while the subject assumed a supine situation at a 50 mm/s speed and a 10 mm/Mv size. ECG recordings and measurements were conducted utilizing CardioLab v. 6.0 General Electric Medical Systems TM.

#### Standard echocardiographic measurements

An experienced echocardiographer performed transthoracic echocardiographic examinations with a cardiac ultrasonography system (Vivid 7®; GE Ving-Med Ultrasound AS; Horten, Norway) equipped with 2.5- to 5-MHz probes. M-mode pulsed and color flow Doppler echocardiographic examinations were performed. Continuous recording of a single-lead ECG was conducted. For all measurements, three cardiac cycles (at least) were evaluated and averaged. Left ventricular end-systolic diameter (LV ESD), LV end-diastolic diameter (LV-EDD), LV ejection fraction (LV EF), interventricular septum thickness (IVS), ascending aortic root diameter (AAR), posterior wall (PW) thickness, left atrial diameter (LAD), and aortic root (AR) diameter were measured according to the guidelines set by the American Society of Echocardiography. Calculation of LV-EF was achieved utilizing Simpson’s technique [20].

The Doppler was employed to analyze the flow velocities of the transmitral pulsed wave. Measurements were taken for the peak flow velocities throughout early diastole (E), late diastole (A), and their ratio (E/A). The Doppler measures were obtained by averaging the results from 3 successive cardiac cycles. A diastolic function designated as normal if the E/A ratio fell between 0.9 and 1.5.

#### Tissue Doppler imaging (TDI)

The measurement of atrial electromechanical conduction was performed using the TDI technique. TDI enables the assessment of myocardial movements, characterized by low speed and high amplitude, in various zones of the heart, with increased temporal resolution. For achieving a good temporal resolution, the width of the sample volume is maintained within the 2 to 5 mm range. The pulsed Doppler sample volume was strategically positioned within specific areas, namely the tricuspid annulus, the lateral mitral annulus, and the septal mitral annulus, within the apical four-chamber view. To achieve the best possible view, the sampled window was carefully aligned to be parallel with the desired myocardial area. Simultaneous TDI and ECG tracing were employed to determine the durations of inter-atrial and intra-atrial electromechanical delays (EMDs). The interval between the P wave onset on the ECG and the tissue Doppler late diastolic peak designates the atrial electromechanical union (PA) time. The calculation of inter-atrial EMD (IA EMD) involved measuring the time disparity from tricuspid PA to lateral PA. The intra-right atrial EMD (RI-EMD) calculation involved measuring time disparity from septal PA to tricuspid PA, while the time disparity from lateral PA to septal PA designates the intra-left EMD (LI-EMD).

#### Liver ultrasonography

Liver ultrasonography was performed for the patients utilizing an Acuson S2000 system (Siemens) with convex and linear transducers (frequency band with 4–14 MHz). Mild steatosis (grade 1) was defined as a subtle and widespread elevation of fine parenchymal echoes, and the portal vein and diaphragm borders being visually normal. Moderate steatosis (grade 2) was described as a moderate and widespread rise of fine echoes, resulting in faintly diminished visibility of the portal vein and diaphragm borders. Severe steatosis (grade 3) was characterized by fine echoes and limited visibility of the diaphragm, portal vein borders, and the right lobe posterior section [21].

#### Anthropometric measurements

Trained physicians performed the necessary measurements of waist circumference (WC), weight, height, and hip circumference (HC) as a part of the anthropometric valuation. The calculation of BMI involved dividing weight (kilograms) via squared height (meters). An automated electronic device (OMRON Model-1 Plus; Omron Company, Kyoto, Japan) was utilized to assess blood pressure at the right arm, with 3 consecutive readings of a 1-min duration after resting in a seated position for at 5 min. The average of these readings was utilized.

#### Laboratory investigations

Blood samples for the 2-h oral glucose tolerance test (OGTT) after overnight fasting were gathered in EDTA-containing tubes. The tubes underwent centrifugation at 4 °C and kept at − 80 °C. An automatic analyzer was used to measure fasting glucose, triglycerides, total cholesterol (TC), glucose levels 2-h post OGTT, low-density lipoprotein (LDL), and high-density lipoprotein (HDL) (Hitachi 7080; Tokyo, Japan). The homeostasis model assessment of insulin resistance (HOMA-IR) was determined as follows: HOMA-IR = fasting serum insulin (mU/mL) × fasting serum glucose (mmol/L) / 22.5. Insulin resistance is denoted by HOMA-IR values ≥ 2.5 [22].

Gamma-glutamyl transferase (GGT), alanine aminotransferase (ALT), aspartate aminotransferase (AST), complete blood count (CBC) including hemoglobin (Hb), hematocrit, white blood cell (WBCs), and platelet count, and highly sensitive C-reactive protein (hs-CRP) were done following standard procedures.

### Statistical analysis

The G*Power program was employed to perform a power analysis, confirming that 97 individuals per group was necessary to attain a 90% power. Mean ± standard deviation (SD) was utilized to express normally distributed quantitative data, where median and range were utilized to express abnormally distributed quantitative data. The categorical variable was expressed using number and percentages. Normality of the data was assessed utilizing the Kolmogorov-Sminrov test. The Student *t*-test was utilized to analyze data sets with a normal distribution, whereas the Mann–Whitney *U* test was employed for abnormally distributed data sets. To assess the association between electromechanical and clinical parameters, Spearman correlation analysis was employed. All analyses were accomplished with SPSS V.20 (SPSS, Chicago. IL, USA). A statistical significance level was established at *P* < 0.05.

## Results

The current study comprises 100 children and adolescents with NAFLD (patient group) and 100 matched healthy children (control group). There were insignificant differences between patient and control groups concerning BMI, SBP, and DBP; however, WC, HC, and W/H ratios were significantly higher in NAFLD children relative to the control group. Likewise, triglyceride levels, ALT, AST, LDL, hs-CRP, and GGT exhibited significantly higher levels in NAFLD children compared to the controls, whereas HDL levels were significantly lower in NAFLD children relative to the controls. All glycemic aspects were significantly elevated in NAFLD children relative to the controls (fasting plasma glucose, 2 h PG, HbA1c, and HOMA-IR) (Table 1).

**Table 1:** Baseline demographic, measurements, clinical data, ultrasonography, and laboratory data of the studied groups

Variable	NAFLD group	Control group	*P* value
Age (years)	15.3 ± 2.5	15.9 ± 2.7	NS
Sex (% male)	48	50	NS
BMI (kg/m^2^)	28.1 ± 5.3	27.9 ± 4.0	NS
SBP (mmHg)	128.2 ± 12.6	125.2 ± 18.4	NS
DBP (mmHg)	81.2 ± 9.6	78.1 ± 10.2	NS
WC (cm)	86.8 ± 11.4	79.1 ± 9.2	< 0.001
HC (cm)	98.3 ± 7.2	89.2 ± 5.9	< 0.001
Waist-hip ratio	0.91 ± 0.14	0.84 ± 0.17	< 0.001
FBG (mmol/L)	6.4 ± 1.3	4.7 ± 1.1	< 0.001
2hPG (mmol/L)	8.5 ± 2.2	7.2 ± 2.4	< 0.001
Triglycerides (mmol/L)	2.4 ± 0.5	1.1 ± 0.6	< 0.001
HbA1c (%)	5.8 ± 0.8	4.9 ± 0.7	< 0.001
HOMA-IR	2.1 (1.81–3.23)	1.47 (1.15–1.77)	< 0.001
LDL (mmol/L)	2.8 ± 0.8	2.4 ± 0.7	< 0.001
HDL (mmol/L)	1.1 ± 0.4	1.4 ± 0.6	< 0.001
AST (units/L)	22.5 ± 12.1	15.4 ± 8.2	< 0.001
ALT (units/L)	64.9 ± 7.3	15.1 ± 10.4	< 0.001
GGT (units/L)	38.2 ± 34.6	22.9 ± 21.7	< 0.001
Hs-CRP (mg/L)	0.6 (0.2–0.9)	0.3 (0.2–0.7)	< 0.001
Steatosis grades: *n* (%)			
Grade I	32		
Grade II	38	-	-
Grade III	30		

*NS* non-significant, *NAFLD* non-alcoholic fatty liver disease, *BMI* body mass index, *SBP* systolic blood pressure, *DBP* diastolic blood pressure, *WC* waist circumference, *HC* hip circumference, *FBG* fasting blood glucose, *2 h PG* postprandial glucose, *HbA1c* glycated hemoglobin, *HOMA-IR* the homeostasis model assessment of insulin resistance, *LDL* low-density lipoprotein, *HDL* high-density lipoprotein, *AST* aspartate aminotransferase, *ALT* alanine aminotransferase, *GGT* γ-glutamyl transferase, *hs-CRP* high sensitive C reactive protein

Atrial electromechanical parameters and echocardiographic properties of the studied groups are listed in Table 2. Compared to the controls, no significant differences have been found in NAFLD patients as regards LV EF, interventricular septal wall, LAD, deceleration time, early and late diastolic wave, HR, or tricuspid PA. The atrial electromechanical parameters measured revealed a notable prolongation of the durations of right and left intra-atrial EMDs as well as the interatrial EMD in NAFLD patients relative to the controls. Moreover, PA lateral and septal times revealed considerable prolongation in the NAFLD patients relative to the control group.

**Table 2:** Atrial electromechanical parameters and echocardiographic characteristics of the participated patients

Variable	NAFLD group	Control group	*P* value
LV-EF (%)	64.2 ± 3.5	65.1 ± 3.6	0.08
LA (cm)	3.2 (2.6–3.8)	3.1 (2.9–3.5)	0.72
IV Septal wall (cm)	0.85 ± 0.49	0.79 ± 0.13	0.68
PW thickness (cm)	0.88 ± 0.38	0.85 ± 0.14	0.13
Deceleration time (ms)	185.2 ± 44.3	177.1 ± 26.3	0.57
E/Em	7.9 ± 2.0	7.3 ± 1.55	0.68
E	75.6 ± 16.1	78.9 ± 8.3	0.47
A	68.5 ± 22.6	69.2 ± 12.9	0.16
HR (beat/min)	75.1 ± 9.5	72.4 ± 10.7	0.34
PA lateral (ms)	64.1 ± 7.2	50.8 ± 5.9	** < 0.001**
PA septal (ms)	41.6 ± 8.2	36.3 ± 6.2	**0.02**
PA tricuspid (ms)	31.8 ± 3.3	30.8 ± 5.0	0.37
IA-EMD (ms)	31.5 ± 7.9	22.04 ± 4.98	**0.01**
RI-EMD (ms)	11.3 ± 4.9	6.93 ± 2.71	** < 0.001**
LI-EMD (ms)	18.2 ± 6.4	15.2 ± 3.8	**0.03**

*NAFLD non-alcoholic fatty liver disease, LV-EF* left ventricular ejection fraction, *LA* left atrium, *PW* posterior wall, *Em* tissue Doppler-derived early diastolic septal wave, *E* early diastolic wave, *A* late diastolic wave, *HR* heart rate, *PA* time duration between the P-wave initiation on surface electrode and the start of the late diastolic wave, *LI-EMD* left intra-atrial electromechanical delay, *RI-EMD* right intra-atrial electromechanical delay, *IA-EMD* interatrial electromechanical delay

In the correlation analysis between a representative of liver enzymes (ALT) and a representative of inflammatory markers (hs-CRP) with echocardiographic and electromechanical parameters (Table 3), there were significant positive correlations with all measured echocardiographic and electromechanical parameters. LV-ejection fraction, early diastolic wave (E), and E/A (late diastolic wave) showed significant negative correlations with both ALT and hs-CRP.

**Table 3:** Correlation between ALT and hs-CRP, with electromechanical parameters and echocardiographic results

Variables	ALT		Hs-CRP	
	*r*	*p*	*r*	*p*
LV-EF (%)	− 0.27	**0.01**	− 0.41	**0.02**
LA (cm)	0.37	** < 0.001**	0.19	** < 0.001**
IV Septal wall (cm)	0.61	**0.02**	0.52	**0.03**
E/Em	0.70	**0.03**	0.66	**0.04**
E	− 0.29	**0.01**	− 0.24	**0.03**
A	0.49	**0.03**	0.51	**0.04**
E/A	− 0.22	**0.02**	− 0.23	**0.01**
PA lateral (ms)	0.25	** < 0.001**	0.41	** < 0.02**
PA septal (ms)	0.49	**0.01**	0.58	**0.01**
PA tricuspid (ms)	0.27	** < 0.001**	0.39	**0.01**
IA-EMD (ms)	0.53	** < 0.001**	0.41	** < 0.001**
RI-EMD (ms)	0.43	** < 0.001**	0.66	** < 0.001**
LI-EMD (ms)	0.40	**0.02**	0.35	**0.02**

*ALT alanine aminotransferase, hs-CRP highly sensitive C-reactive protein, LV-EF* left ventricular ejection fraction, *LA* left atrium, *Em* tissue Doppler-derived early diastolic septal wave, *E* early diastolic wave, *A* late diastolic wave, *HR* heart rate, *PA* time elapsed between the P-wave initiation on surface electrocardiogram and the late diastolic wave commencement, *LI-EMD* left intra-atrial electromechanical delay, and *RI-EMD* right intra-atrial electromechanical delay, *IA-EMD* interatrial electromechanical delay

## Discussion

This study is believed to be the first prospective study conducted to explore the linkage between the risk of AF development and NAFLD in pediatrics. The present study revealed that children with NAFLD showed atrial electromechanical alterations that may presage AF in adulthood. Similar observations have been made in studies involving adults, where they demonstrated an elevated AF incidence and prevalence among adults with NAFLD [23–25]. Epidemiological evidence is accumulating, establishing NAFLD as a significant contributor to AF development with AF prevalence ranging from 10 to 15% in patients with NAFLD [3, 5]. Given the rising global prevalence of NAFLD, it is reasonable to anticipate a future increase in the frequency and potential occurrence of AF among individuals with NAFLD.

Minhas et al. [26] investigated the linkage between AF and NAFLD in adults through a meta-analysis and systematic review. They observed a statistically significant elevation in the AF risk within NAFLD patients across all the analyzed studies, and this association remained significant in various subgroup analyses. While observation-based studies cannot establish causal relationship, the evaluated studies fulfilled several criteria proposed by Hill (1965) [27] for establishing causation. Firstly, a considerable linkage between NAFLD and AF was noticed, which is unlikely to have occurred by chance. Secondly, consistency was observed across different studies, as the relationship was noted in diverse populations and geographical locations. Thirdly, two of the evaluated studies revealed a distinct temporal connotation [14], where NAFLD preceded the AF onset. Lastly, there exists a plausible biological explanation for the incorporation of NAFLD in AF development. These aspects collectively suggest a potential causal relationship.

Our study revealed significantly higher WC, HC, WHR, HOMA-IR, and hs-CRP in patients with NAFLD compared to the control group. Other studies propose that IR, visceral obesity, and the inflammatory environment associated with NAFLD likely contribute to AF emergence [28, 29]. Increased risk of AF revealed to be linked to systemic inflammation presented by elevated CRP [30].

Moreover, our study revealed significantly higher ALT, AST, and GGT in patients with NAFLD compared to the control group. Interestingly, Sinner et al. [25] evaluated 3744 AF patients over 10 years, and the researchers found that elevated levels of serum transaminases (regardless of the cause of liver disease) were independent risk factors for AF.

The precise pathophysiologic mechanisms underlying the propensity of NAFLD patients in developing AF are not yet fully understood. However, the presence of common risk factors between the two conditions such as diabetes, hypertension, and obesity may be one of the mechanism [14]. Additionally, NAFLD patients often exhibit cardiac structural alterations that could predispose them to AF [31]. Several studies have documented enlarged left ventricular wall mass and thickness, left atrial size and volume, and reduced diastolic function in NAFLD individuals, which are significant contributor to AF in adults [32, 33]. A recent large study involving more than 3000 middle-aged individuals diagnosed with NAFLD via CT scans revealed an independent linkage among subclinical myocardial dysfunction between cardiac remodeling and NAFLD, regardless of recognized incident factors for heart failure [31]. Therefore, it is reasonable to consider that early myocardial remodeling in the presence of NAFLD could possibly impact cardiac function and metabolism, thereby increasing the likelihood of developing AF.

Moreover, NAFLD is linked to autonomic dysfunction [34, 35] characterized by disrupted sympathovagal stimulation, which is linked to an elevated AF risk [36]. Furthermore, liver fibrosis may significantly contribute to cardiac remodeling and AF development in NAFLD. A recent cross-sectional analysis revealed a strong association between pre-clinical hepatic fibrosis, evaluated via T1-mapping MRI, and prevalent AF. There was a correlation observed between liver stiffness indices derived from T1 mapping and both structural and functional parameters of the heart, including myocardial fibrosis, left atrial dysfunction, and left ventricular hypertrophy, which may partially explain the mechanisms underlying the coincidence of AF with liver fibrosis [37].

Another possible mechanism is that obesity and NAFLD cause an increase in the inflammatory mediators and oxidative stress [38]. Both AF and NAFLD progress as a result of cell death and the fibrosis that follows from oxidative radicals and inflammatory infiltration. AF is caused by changes in cardiac myocytes that are both structural (fibrosis) and cellular (ion current modification) [39]. Ozveren et al. revealed a longer interatrial electromechanical delay and conduction abnormality in NAFLD patients after adjusting for heart disease and hypertension [32]. This is caused by cellular ion channelopathies and increased fibrosis in the cardiomyocytes of patients with NAFLD. This theory is going with our results as increased electromechanical parameters were positively correlated with hs-CRP as a marker of inflammation in pediatric patients with NAFLD.

The results of our study demonstrate an increase in inter-atrial and intra-atrial conduction, which could presage the appearance of AF later in adulthood. Hence, early prevention and treatment of NAFLD in childhood could potentially preclude the onset of AF in later life. Further longitudinal follow-up is necessary to identify the actual development of AF and its outcomes.

### Limitations of the study

No follow-up of the cases for the occurrence of AF as most of research reported patients with NAFLD develop AF later on in adulthood that need long follow-up. Moreover, we did not assess the presence of atrial fibrous tissue by MRI.

## Conclusion

Children with NAFLD show atrial electromechanical alterations that may presage the appearance of AF in adulthood. These children require multidisciplinary medical care to control liver disease and cardiovascular complications.

## Data Availability

All the data of the study are available from the corresponding author on reasonable request.

## Abbreviations

AF: Atrial fibrillation
ALT: Alanine aminotransferase
AST: Aspartate aminotransferase
DBP: Diastolic blood pressure
Em: Tissue Doppler-derived early diastolic septal wave
FBG: Fasting blood glucose
GGT: γ-Glutamyl transferase
HbA1c: Glycated hemoglobin
HDL: High-density lipoprotein
HOMA-IR: The homeostasis model assessment of insulin resistance,
HR: Heart rate
hs-CRP: Highly sensitive C-reactive protein
IA-EMD: Interatrial electromechanical delay
IL: Interleukin
LA: Left atrium
LDL: Low-density lipoprotein
LI-EMD: Left intra-atrial electromechanical delay
LV-EF: Left ventricular ejection fraction
NAFLD: Non-alcoholic fatty liver disease
PA: Time elapsed between the P-wave initiation on surface electrocardiogram and the late diastolic wave commencement
PW: Posterior wall
RI-EMD: Right intra-atrial electromechanical delay
SBP: Systolic blood pressure

## Code availability

Not applicable

all the data that of the study are available from the corresponding author on reasonable request.
